# Investigation of Circulating Extracellular Vesicle MicroRNA Following Two Consecutive Bouts of Muscle-Damaging Exercise

**DOI:** 10.3389/fphys.2018.01149

**Published:** 2018-08-20

**Authors:** Jason A. C. Lovett, Peter J. Durcan, Kathryn H. Myburgh

**Affiliations:** Department of Physiological Sciences, Stellenbosch University, Stellenbosch, South Africa

**Keywords:** extracellular vesicles, exosomes, microRNAs, blood samples, skeletal muscle, exercise, eccentric contraction, myomiR

## Abstract

**Background:** Extracellular vesicles (EVs) are nano-sized vesicles that are known to be powerful mediators of intercellular communication via their microRNA (miR) content. A paucity of information on EV-mediated communication arising from skeletal muscle (SkM) in response to exercise-induced muscle damage is present in the published literature. Lack of such information inhibits our understanding of muscle injury and repair processes.

**Aims:** To assess circulating EV levels and selected miR content within them, in response to two consecutive bouts of muscle-damaging exercise.

**Methods:** Serum creatine kinase activity (CK) and EVs were analyzed from the blood of 9 healthy, untrained males at baseline, and at 2 and 24 h post-exercise. The exercise regimen consisted of a combination of plyometric jumping and downhill running. Perceived muscle pain (PMP) was assessed on a scale from 1 to 10. Plasma EVs were isolated using size exclusion columns and visualized with transmission electron microscopy (TEM). EV size and number were quantified using nanoparticle tracking analysis (NTA). miR expression was quantified using qPCR, with normalization to an exogenous control (cel-miR-39).

**Results:** PMP and CK were significantly elevated post-exercise compared to baseline levels, providing indirect evidence for muscle damage. EV visualization using TEM revealed an abundant and heterogeneously sized pool of intact particles within the exosome size range (30–150 nm). No significant change in mean EV size or number was seen over time. The SkM-specific miR-206 in EVs was found to be variable among participants and no significant change occurred in SkM-important miRs; 1, 133a, 133b, 486, and 499a. However, EV miR-31 decreased from baseline to 24 h post-exercise (*p* = 0.027).

**Conclusion:** Mild to moderate exercise-induced muscle damage altered the miR-31 profile of circulating EVs within the first 24 h post-exercise, but not that of myomiRs in EVs. These data demonstrate that EVs carry selectively packaged cargo which can be affected by exercise. Future research into the total miR content of EVs in response to exercise-induced muscle damage may reveal other miRs responsive to this relatively mild perturbation. More time points post-muscle-damaging exercise would provide a better understanding of the temporal EV myomiR response.

## Introduction

The regeneration of skeletal muscle (SkM) following injury is a complex yet vital physiological process. Divergent cell types such as immune, endothelial and satellite cells must synchronize communication to ensure regeneration occurs effectively. The molecular mechanisms that underpin such a system of communication remain largely unclear. In the past decade a novel mode of intercellular communication has emerged in the form of extracellular vesicles (EVs) (Record et al., 2014; Ratajczak and Ratajczak, 2016) . EVs are nano-sized (30–1,000 nm), selective mediators of intercellular communication (Skotland et al., 2017; Xie et al., 2017). Structurally, EVs are comprised of a spherical, cell-like lipid bilayer that encapsulates and shuttles biomolecules (i.e., lipids, proteins and RNA) between cells (Yáñez-Mó et al., 2015). Importantly, EVs are abundant in biofluids (Andreu et al., 2016), suggesting their potential as mediators of inter-organ, distant communication. Their ubiquitous presence and their ability to be both secreted and internalized by mammalian cells (Ekström et al., 2013; Forterre et al., 2014a; Madison et al., 2014) highlights the need for more information on how EVs may play a role in complex physiological processes such as SkM regeneration.

Of the biomolecules transferred by EVs, RNA, and microRNA (miR) in particular, have received the most attention from the scientific community. miRs are small, non-coding RNAs that transiently repress the translation of mRNA (Filipowicz et al., 2008). Their potent regulation of translation enables miRs to have powerful effects on cells that can be as substantial as the alteration of the recipient cell’s phenotype (Lim et al., 2005). The encapsulation of miRs within EVs allows for their protection from abundant RNases as they travel through the circulatory system (Kosaka et al., 2010; Shelke et al., 2014), which thus promotes distant yet powerful communication between cells/organs.

Thousands of miRs have been reported to be present within EVs isolated from human blood (Huang et al., 2013; Lugli et al., 2015; Garcia-Contreras et al., 2017; Xie et al., 2017). Both *in vivo* and *in vitro* studies have shown that miRs are selectively packaged into EVs (Forterre et al., 2014b; Guescini et al., 2015; Garcia-Contreras et al., 2017). Such a scenario highlights the importance of studying the miR content of circulating EVs in human biofluids to better understand potential cross-talk between cells/organs.

Skeletal muscle is the largest internal organ of the human body, and it is therefore likely that EVs secreted from this tissue form a large part of the total circulating EV profile during normal homeostasis. Perturbation of SkM, such as from the occurrence of injury in response to exercise, may alter circulating EV profiles and/or their miR content.

Analysis of circulating myomiRs (i.e., muscle-enriched miRs that are expressed in ≥20-fold abundance then their average abundance found in other tissues (Lee et al., 2008; McCarthy, 2011) in plasma/serum in response to exercise is not an entirely new concept. The majority of previous studies did not focus on EV miR content specifically, but rather, focused on the exercise response of total circulating miRs (c-miRs) extracted from whole plasma/serum (Baggish et al., 2011; Banzet et al., 2013; Uhlemann et al., 2014). However, the miR profile between whole plasma/serum and EVs has recently been reported to differ, suggesting that it is important to measure EV miR content (Xie et al., 2017). There is currently a paucity of information available in the published literature investigating EV miR content in response to exercise. To the best of the authors’ knowledge, no study has yet investigated any potential changes in circulating EV miR profiles in response to muscle-damaging exercise.

To investigate whether exercise-induced muscle damage can alter the circulating EV profile, we analyzed the number and size of EVs, as well as the expression of selected miRs within EVs pre- and post-two consecutive bouts of muscle-damaging exercise [i.e., plyometric jumping (PMJ) and downhill running (DHR)]. These exercise modalities have been shown previously by our research group to induce muscle damage in untrained individuals (Van De Vyver and Myburgh, 2012; Macaluso et al., 2014). The muscle response to damaging exercise is a process that evolves over a period of time post-exercise (Van De Vyver and Myburgh, 2012). Banzet et al. (2013) have previously indicated that selected c-miRs are significantly altered 2–6 h post-downhill walking. Therefore the time points 2 and 24 h post muscle-damaging exercise were selected for this study.

A total of 8 miRs; miR-1, 31, 133a, 133b, 206, 208b, 486, and 499a were selected for investigation with regards to their expression levels in EVs. All 8 miRs have been reported to be involved in the growth and/or development of SkM (Chen et al., 2006; Rosenberg et al., 2006; Mccarthy, 2008; Van Rooij et al., 2009; Small et al., 2010; Crist et al., 2012). Seven of the 8 miRs (miR-1, 133a, 133b, 206, 208b, 486, and 499a) investigated are myomiRs. The additional miR selected for investigation, miR-31, is expressed across many tissues, and is therefore not classified as a myomiR. However, miR-31 was included in the current study due to its proposed importance in maintaining quiescence of local SkM progenitor cells, satellite cells (Crist et al., 2012). This is a cell type important for the regeneration of muscle in mammals (Lepper et al., 2011).

Our results provide empirical support that the levels of miR-31 are altered in circulating EVs during the early-phase response to muscle damage. Size and number of EVs were not found to be altered significantly, which highlighted the importance of looking within EVs to assess biological cargo content.

## Materials and Methods

### Participants

Nine healthy, untrained males between the age of 18 and 30 took part in this study. Exclusion criteria included; regular exercise exceeding 2 bouts per week, participation in professional sports, or the use of anti-inflammatory medication within 3 months preceding the study. Furthermore, participants were required to be in good health and were not to have participated in any strenuous exercise in the 3 weeks preceding the study. This study was approved by Stellenbosch University’s Health Research Ethics Committee and was carried out in accordance with the guidelines of the declaration of Helsinki.

### Muscle-Damaging Exercise Regimen

Each participant performed two consecutive bouts of muscle-damaging exercise comprising a combination of PMJ and DHR. The protocol for both modes of exercise were modified from similar protocols previously used in our lab (Van De Vyver and Myburgh, 2012; Macaluso et al., 2014). Briefly, participants arrived at the exercise lab at 10 am, and were guided through a 5 min warm-up regimen that focused on the quadriceps muscles. Following this, all participants were required to complete 10 sets of 10 plyometric jumps at 90% of their maximum achievable jump height, with a 1 min interval between sets. Following this, a 5 min rest period was given before commencement with the DHR regimen. Here, participants were required to perform 5 sets of 4 min bouts of DHR at a speed of 10 km/h. The treadmill was set at a 10% decline, and a 2 min standing interval between sets was given.

### Indirect Indicators of Muscle Damage

Whole blood was drawn from the antecubital vein by a qualified phlebotomist at baseline, and at 2 and 24 h post-exercise. For standardization, participants were given a standard meal replacement shake post-exercise and requested not to eat until they returned for the 2 h blood draw. Additionally, participants were requested not to take part in strenuous activity until they returned the next day for a 24 h blood draw.

Plasma was isolated from whole blood and stored for subsequent EV isolation. Briefly, blood was collected into EDTA-coated tubes and centrifuged at 1,200 ×*g* for 10 min at 4°C. Plasma was aliquoted and immediately frozen at -80°C. An additional aliquot of whole blood was collected into heparin-coated tubes for serum creatine kinase analysis (analysis done by PathCare, South Africa). Additionally, at 2, 24, and 48 h post-exercise, participants gave a rating of their perceived muscle soreness, out of 10, upon walking. A score of 0 signified no pain, with one corresponding to slight pain and 10 signifying extreme pain.

### Extracellular Vesicle Isolation

Extracellular vesicles were isolated from plasma using qEV size exclusion columns (SEC) (iZon Science) as per the manufacturer’s protocol. qEV columns result in a predominantly exosome isolate, with microvesicles appearing infrequently (Lobb et al., 2015). Plasma was thawed on ice and centrifuged at 15,000 ×*g* for 10 min to remove cellular debris. One milliliter of plasma was then added to the top of the column, and multiple 500 μl fractions of the resultant eluent were collected. Fractions 7–9 were pooled due to their known high vesicle number and purity (see qEV column white paper)^1^. Samples were aliquoted according to the volume required for subsequent analysis, and frozen at -80°C.

### Transmission Electron Microscopy

Two hundred-mesh, carbon-coated copper TEM grids (Electron Microscopy Sciences, United States) were glow-discharged and incubated on 10 μl of sample for 10 min. Grids were then washed with dH_2_O and carefully dabbed onto Whatman paper, and subsequently incubated atop 10 μl of filtered 2% uranyl acetate for contrast. Grids were viewed on a Phillips Tecnai TEM.

### MicroRNA Analysis

Total RNA was isolated from 600 μl of EV isolate (i.e., pooled Fractions 7–9) using the Total Exosome RNA and Protein isolation kit as per the manufacturer’s guidelines (Thermo Fisher Scientific, Waltham, MA, United States; 4478545). Samples were immediately frozen at -80°C. Samples were thawed on ice and reverse transcribed using the TaqMan Advanced miRNA cDNA synthesis kit (Thermo Fisher Scientific, Waltham, MA, United States; A28007). qPCR analysis was achieved using TaqMan Advanced miRNA assays; miR-1 (477820_mir), miR-31 (478015_mir), miR-133a (478511_mir), miR-133b (480871_mir), miR-206 (477968_mir), miR-208b (477806_mir), miR-486 (478128_mir), and miR-499a (478561_mir) (all obtained from Thermo Fisher Scientific, Waltham, MA, United States). Five micro liter of cDNA was loaded into fast optical 96-well plates and sealed with optical adhesive film. qPCR analysis was performed in duplicates on a StepOne qPCR thermocycler (Applied Biosystems).

Additionally, 1 pM of the synthetic C. Elegans oligo, cel-miR-39 (Sequence: UCACCGGGUGUAAAUCAGCUUG), was added to the isolated total RNA. This sequence does not exist in humans and was used as an exogenous control. All qPCR reactions were normalized to their corresponding cel-miR-39 *C*_t_ values.

### Nanoparticle Tracking Analysis

Particle size and number were analyzed with nanoparticle tracking analysis (NTA), using the NanoSight NS500 (Malvern Instruments). Analysis was performed at the Council for Scientific and Industrial Research (CSIR, Pretoria, South Africa). EV isolates were diluted in PBS (1:200) to achieve a suitable number of particles per frame, and five 30 s videos were used to determine particle numbers per ml, as well as mean particle diameters.

### Statistical Analysis

All data are represented as mean ± standard error of the mean (SEM). Parametric data were analyzed by means of a one-way ANOVA, with a Fisher LSD *post hoc* test. A mixed-model, one-way ANOVA was used for matched data with missing data points. MicroRNA *C*_t_ values were normalized to an exogenous control, cel-miR-39, by subtracting the sample from the control *C*_t_. Graphs were made using version 5 of GraphPad Prism (GraphPad Software Inc., United States), and statistical significance was set at a 5% confidence interval, i.e., *p* < 0.05.

## Results

### Participants

Nine healthy male participants between the age of 18 and 30 successfully completed the combined exercise protocol. Mean participant height and weight were 76 ± 4 kg and 179 ± 1.86 cm, respectively.

### Indirect Markers of Muscle Damage

**FIGURE 1:**
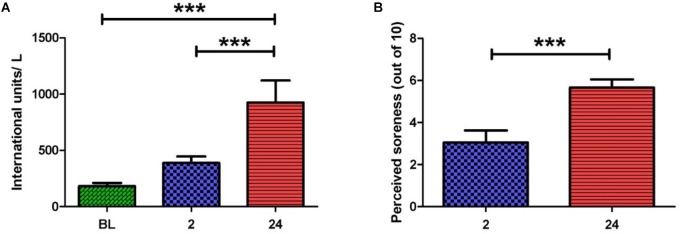
**(A)** Serum creatine kinase activity before and after the exercise intervention. Values are represented in international units (IU) per liter. A large increase in scores is seen at 2 and 24 h post-exercise. **(B)** Perceived muscle pain of the anterior quadriceps upon walking. A large increase in pain scores is seen at 24 h when compared to 2 h post-exercise (one-way ANOVA. Mean ± SEM; ^∗∗∗^*p* < 0.001; *n* = 9).

Serum CK activity is an indirect marker of muscle damage (Clarkson and Hubal, 2002) and hence was measured at baseline (BL), 2 and 24 h post-exercise (**Figure 1A**). When compared to BL, CK activity was significantly elevated at 24 h post-exercise (925.8 ± 196.2 vs. 182.0 ± 29.0 IU/L, *p* < 0.001). A significant difference in CK activity was also observed between 2 and 24 h post-exercise (388.7 ± 57.0 vs. 925.8 ± 196.2 IU/L, *p* < 0.001), whereas no change was noted between BL and 2 h post-exercise.

Participants rated their perceived muscle pain (PMP) in the anterior quadriceps upon walking. Ratings were obtained at 2, 24, and 48 h post-exercise (**Figure 1B**). PMP during walking was significantly higher at 24 and 48 h when compared to 2 h post-exercise (5.7 ± 0.8 vs. 2.7 ± 0.5, *p* < 0.001) and (5.4 ± 0.7 vs. 2.7 ± 0.5, *p* < 0.05). The PMP scores observed here are in alignment with the known timeframe for delayed onset muscle soreness manifestation (i.e., 24–48 h post-exercise). Taken together, PMP and CK aided our confirmation that muscle damage had indeed taken place.

### Extracellular Vesicle Characterization

**FIGURE 2:**
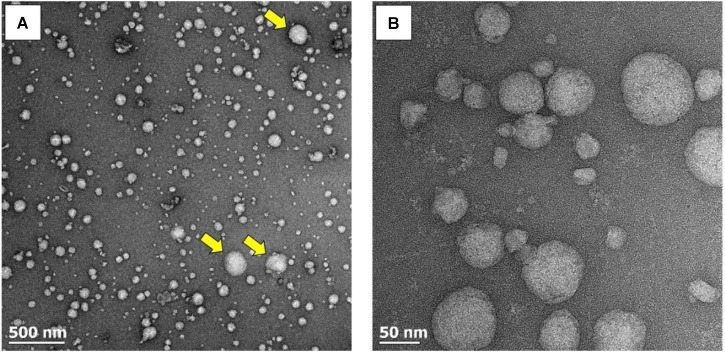
TEM images of EVs at increasing magnifications. **(A)** A wide-field image revealing the abundance of EVs. EVs within the exosome size range (30–150 nm) predominate, with microvesicles (100–l,000 nm) appearing less frequently (yellow arrows). **(B)** Highly resolved images of small exosome-sized particles.

The qEV SEC utilized in this study have previously been used to isolate EVs from human plasma (Lobb et al., 2015; Takov et al., 2017). To confirm the presence of EVs in the pooled fractions (i.e., fractions 7–9) obtained from plasma pre and post-exercise, an aliquot of the isolated fractions was visualized using TEM. An abundance of particles with the expected diameter and morphology of EVs was present (**Figure 2A**). The spherical appearance of these particles suggests that they were isolated in an intact state and had not been lysed by the isolation or fixation process. Qualitative assessment of TEM images suggested that the majority of isolated vesicles fell within the exosome size range (30–150 nm), with vesicles in the microvesicle size range (100–1,000 nm) appearing less frequently (**Figure 2A** yellow arrows). Highly resolved visualization of ± 50 nm exosome-sized particles was also achieved (**Figure 2B**).

**FIGURE 3:**
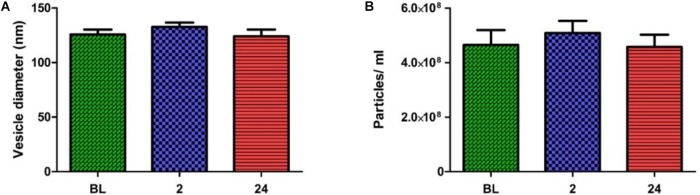
Nanoparticle tracking analysis results across all time points. No change in particle diameters **(A)** or particles/ml **(B)** were noted over time (one-way ANOVA. Mean ± SEM; *n* = 9 for BL and 24 h; *n* = 8 for 2 h).

In order to obtain quantitative data on the number and size of EVs obtained using the SEC method, samples were analyzed with NTA. The pooled fractions were abundant in vesicles within the exosome size range (i.e., 30—150 nm). No significant change in vesicle number or size was seen over time (**Figures 3A,B**). The combined results of TEM and NTA demonstrate that successful isolation of EVs from human plasma had been achieved, with the exercise intervention having no effect on physical EV dynamics.

### MicroRNA Cargo Analysis

**FIGURE 4:**
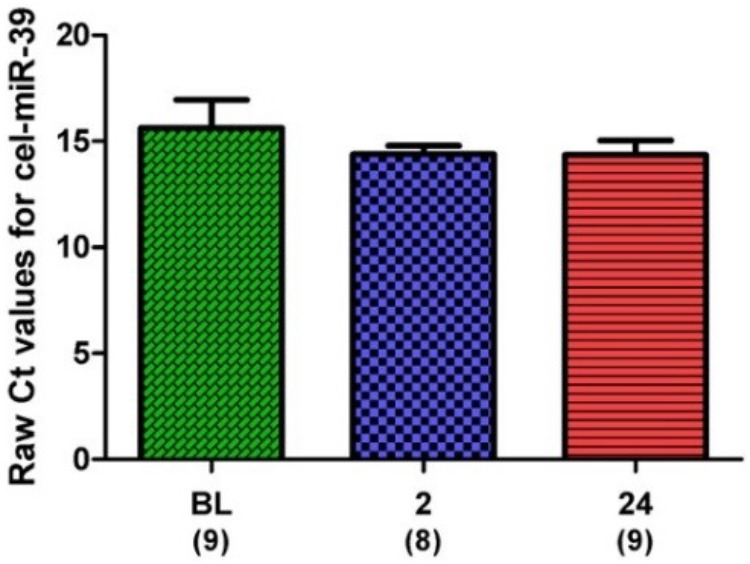
Raw *C*_t_ values for the spike-in control, cel-miR-39. Samples for all time points are represented as Mean ± SEM.

microRNA expression was normalized to an exogenous control (cel-miR-39) and expressed as Δ*C*_t_ (i.e., sample – control). Expression of the exogenous control across all samples and time points is shown in **Figure 4**.

**FIGURE 5:**
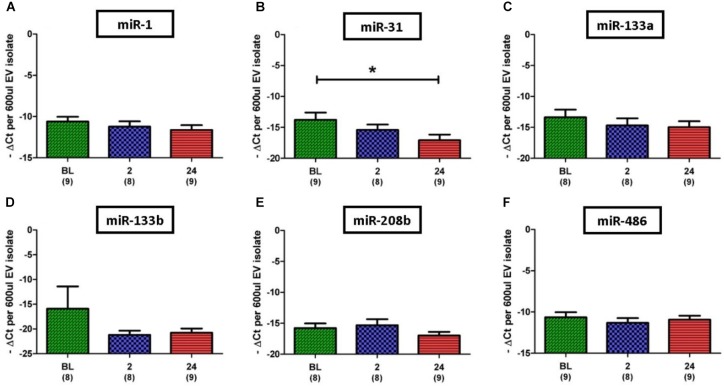
Expression of six EV miR levels normalized to the exogenous control, cel-miR-39. **(A,C–F)** Relative expression of myomiRs, with no changes noted over time. **(B)** Decrease in miR-31 at 24 h when compared to BL. Number of subjects for each analysis is noted below the time point. Data are represented as -Δ*C*_t_ per 600 μl EV isolate. An arbitrary negative is assigned to each sample so that samples with lower miR content and thus a higher *C*_t_ will be deemed to have a lower bar graph (one-way ANOVA. Mean ± SEM; ^∗^*p* < 0.05).

Based on results regarding SkM in the published literature, eight miRs were selected for investigation in this study; miR-1, 31, 133a, 133b, 206, 208b, 486, and 499a. Initial analysis of the miR data demonstrated that expression levels of the SkM-specific, miR-206, and miR-499a among individuals and even between time points for the same individual were very diverse. In one participant for example, expression of miR-206 was not detectable at any time point, whilst for another expression was detectable at every time point. Due to such variation we omitted miR-206 and miR-499a from statistical analysis. In contrast to miR-206 and miR-499a, miR-1 was the most abundantly expressed miR, with strong expression (*C*_t_ < 30) detected in all but one of 26 samples. Quantitative analysis of miR expression levels showed no significant difference between time points for miR-1, 133a, 133b, 206, and 486 (**Figures 5A,C–F**). However, expression of miR-31 was found to be significantly lower at 24 h post-exercise when compared to BL (13.8 ± 1.2 vs. 17.1 ± 0.9 Δ*C*_t_, *p* = 0.027) (**Figure 5B**).

## Discussion

An acute bout of muscle-damaging exercise is known to stimulate circulating immune cells and produce elevated circulating levels of the SkM damage marker, CK (Clarkson et al., 1987; Clarkson and Hubal, 2002; van de Vyver and Myburgh, 2014). However, whether changes in circulating EVs occur after muscle-damaging exercise is not known. Here we show for the first time that two consecutive bouts of muscle-damaging exercise is capable of changing the miR profile of circulating EVs in humans, albeit selectively.

Our investigation of 8 miRs in response to exercise-induced muscle damage showed that the presence of one miR in circulating EVs, miR-31, is significantly reduced 24 h post-performance of a mildly muscle-damaging exercise bout when compared to baseline levels. While the physiological importance of such a decrease remains unclear it is noteworthy that miR-31 is known to transiently suppress the translation of the mRNA of the satellite cell activator Myf5 (Crist et al., 2012). Such suppression allows satellite cells to maintain a quiescent state. Satellite cells are endogenous SkM stem/progenitor cells that are essential for SkM repair following injury (Lepper et al., 2011). Usually satellite cells, like most adult stem cells, are found in a quiescent state and hence require a signal to become activated. Notably, our research group has previously found that satellite cell activation in humans peaks at 24 h post-exercise when an exercise intervention similar to the one here is performed (Van De Vyver and Myburgh, 2012). Based on the difference in satellite cell numbers comparing individuals who exercise regularly versus those who are sedentary, Macaluso and Myburgh (2012) previously suggested that circulating factors affected by exercise could control basal levels of progenitor cell activation. The previous findings and the current findings combined suggest that if satellite cells take up circulating EVs as most mammalian cells are known to, then EVs may be involved in the control of satellite cell quiescence versus activation status. Provision of miR-31 on a regular basis would maintain quiescence. Removal of this inhibitory regulation of Myf5 translation by miR-31 via decreased donation of miR-31 to satellite cells by EVs containing less miR-31 would conversely allow satellite cells to become activated.

The variability in miR-206 detection observed here mirrors that found by Banzet et al. (2013), who investigated miR-206 in whole plasma following downhill walking exercise. Here, in samples that had no detectable miR-206, detectable levels of other miRs such as miR-1, 31, 133a, 133b, 208b, and 486 were present. This suggests that miR-206 may be more selectively packaged into EVs than other miRs, or display a different temporal profile. The combined findings of variable miR-206 levels in both whole plasma and EVs is important for future studies that seek to analyze circulating miR-206 expression in humans, as greater numbers of participants may be required in different intervention groups to account for such variability.

Researchers have only recently begun to assess the profiles of circulating EVs in differing physiological/pathological states, yet clear correlations between either disease types or stage of disease has emerged (Melo et al., 2015; Koutsoulidou et al., 2017; Skotland et al., 2017). In this study, we used two indirect markers of muscle damage, CK and PMP, to confirm the occurrence of the physiological stressor, SkM damage. An over fivefold increase in CK was seen between baseline and 24 h. This large increase occurred with a concomitant increase in PMP. Taken together, these data confirmed that perturbation of the non-injured physiological state had occurred and hence reinforce the premise that the responses observed in this study in circulating EV miR-31 may indeed be relevant to muscle-damaging exercise.

The combined exercise modalities utilized in this study were PMJ and DHR, and were implemented in order to maximize the potential for mild exercise-induced muscle damage to occur. Both of these modalities have been shown by our research group to induce mild muscle damage in humans (Macaluso et al., 2014; van de Vyver and Myburgh, 2014), and therefore our intervention is a useful model for the study of changes in circulating EVs post-mildly muscle-damaging exercise. However, it should be noted that the DHR portion of the intervention used here has a relatively large aerobic component, and therefore the observations cannot be solely attributed to SkM tissue damage. Previously it has been shown that choice of exercise modality does appear to influence circulating miRs in plasma (Banzet et al., 2013). Therefore, pure eccentric/muscle-damaging exercise should be considered in the future, in order to remove any influence of aerobic exercise.

We did not observe any significant change in particle size or number 2 or 24 h post-exercise compared to baseline levels. Notably, different exercise modalities, e.g., aerobic (Kramers, 2015) or muscle-damaging (our study) appear to have no major impact on circulating EV number when EV numbers are assessed more than 1hr post-performance of the exercise bout. However, if EV number is assessed immediately post-performance of an aerobically based exercise intervention a significant increase in EV number has been reported (Kramers, 2015). We did not assess EV number immediately post the muscle-damaging exercise intervention and hence whether the same temporal increase occurs with muscle-damaging exercise remains to be determined.

## Conclusion

The most significant finding of the current study was that the miR-31 cargo of circulating EVs significantly decreased by 24 h post-performance of mild to moderate muscle-damaging exercise, in the form of PMJ followed by DHR, when compared to baseline levels. This finding supports the need for more investigation into the role circulating EVs may play in SkM regeneration, as miR-31 is a known regulator of translation for the satellite cell activator Myf5. A more comprehensive miR analysis of EV content that not only focuses on myomiRs may yet yield some additional targets to explore for a better understanding of responses of local and distant stem cells to muscle-damaging exercise.

## Ethics Statement

This study was carried out in accordance with the recommendations of the Declaration of Helsiniki. The protocol was approved by the Health Research Ethics Committee of Stellenbosch University. All subjects gave written informed consent in accordance with the Declaration of Helsinki.

## Author Contributions

JL conceived and designed the experiments, performed the experiments, analyzed the data, and wrote the manuscript. PD analyzed the data and edited the manuscript. KM conceived and designed the experiments, analyzed the data, and edited the manuscript.

## Conflict of Interest Statement

The authors declare that the research was conducted in the absence of any commercial or financial relationships that could be construed as a potential conflict of interest. The reviewer DVP and handling Editor declared their shared affiliation.

## Footnotes

1 http://izon.com/wp-content/uploads/qEV-WP-1.pdf

## References

[B1] Andreu Z. Rivas E. Sanguino-Pascual A. Lamana A. Marazuela M. González-Alvaro I. (2016). Comparative analysis of EV isolation procedures for miRNAs detection in serum samples. *J. Extracell. Vesicles* 5:31655. 10.3402/jev.v5.31655 27330048 PMC4916259

[B2] Baggish A. L. Hale A. Weiner R. B. Lewis G. D. Systrom D. Wang F. (2011). Dynamic regulation of circulating microRNA during acute exhaustive exercise and sustained aerobic exercise training. *J. Physiol.* 589 3983–3994. 10.1113/jphysiol.2011.213363 21690193 PMC3179997

[B3] Banzet S. Chennaoui M. Girard O. Racinais S. Drogou C. Chalabi H. (2013). Changes in circulating microRNAs levels with exercise modality. *J. Appl. Physiol.* 115 1237–1244. 10.1152/japplphysiol.00075.2013 23950168

[B4] Chen J. F. Elizabeth M. Mandel J. Thomson M. Wu Q. Hammond S. M. (2006). The role of microRNA-1 and microRNA-133 in skeletal muscle proliferation and differentiation. *Nat. Genet.* 38 228–233. 10.1038/ng1725 16380711 PMC2538576

[B5] Clarkson P. M. Byrnes W. C. Gillisson E. Harper E. (1987). Adaptation to exercise-induced muscle damage. *Clin. sci.* 73 383–386. 10.1042/cs0730383 3665359

[B6] Clarkson P. M. Hubal M. J. (2002). Exercise-induced muscle damage in humans. *Am. J. Phys. Med. Rehabil.* 81 S52–S69. 10.1097/00002060-200211001-00007 12409811

[B7] Crist C. G. Montarras D. Buckingham M. (2012). Muscle satellite cells are primed for myogenesis but maintain quiescence with sequestration of Myf5 mRNA targeted by microRNA-31 in mRNP granules. *Cell Stem Cell* 11 118–126. 10.1016/j.stem.2012.03.011 22770245

[B8] Ekström K. Omar O. Granéli C. Wang X. Vazirisani F. Thomsen P. (2013). Monocyte exosomes stimulate the osteogenic gene expression of mesenchymal stem cells. *PLoS One* 8:e75227. 10.1371/journal.pone.0075227 24058665 PMC3776724

[B9] Filipowicz W. Bhattacharyya S. N. Sonenberg N. (2008). Mechanisms of post-transcriptional regulation by microRNAs: are the answers in sight? *Nat. Rev. Genet.* 9 102–114. 10.1038/nrg2290 18197166

[B10] Forterre A. Jalabert A. Berger E. Baudet M. Chikh K. Errazuriz E. (2014a). Proteomic analysis of C2C12 myoblast and myotube exosome-like vesicles: a new paradigm for myoblast-myotube cross talk. *PLoS One* 9:e84153. 10.1371/journal.pone.0084153 24392111 PMC3879278

[B11] Forterre A. Jalabert A. Chikh K. Pesenti S. Euthine V. Granjon A. (2014b). Myotube-derived exosomal miRNAs downregulate sirtuin1 in myoblasts during muscle cell differentiation. *Cell Cycle* 13 78–89. 10.4161/cc.26808 24196440 PMC3925739

[B12] Garcia-Contreras M. Shah S. H. Tamayo A. Robbins P. D. Golberg R. B. Mendez A. J. (2017). Plasma-derived exosome characterization reveals a distinct microRNA signature in long duration type 1 diabetes. *Sci. Rep.* 7:5998. 10.1038/s41598-017-05787-y 28729721 PMC5519761

[B13] Guescini M. Canonico B. Lucertini F. Maggio S. Annibalini G. Barbieri E. (2015). Muscle releases alpha-sarcoglycan positive extracellular vesicles carrying miRNAs in the bloodstream. *PLoS One* 10:e0125094. 10.1371/journal.pone.0125094 25955720 PMC4425492

[B14] Huang X. Yuan T. Tschannen M. Sun Z. Jacob H. Du M. (2013). Characterization of human plasma-derived exosomal RNAs by deep sequencing. *BMC Genomics* 14:319. 10.1186/1471-2164-14-319 23663360 PMC3653748

[B15] Kosaka N. Iguchi H. Yoshioka Y. Takeshita F. Matsuki Y. Ochiya T. (2010). Secretory mechanisms and intercellular transfer of microRNAs in living cells. *J. Biol. Chem.* 285 17442–17452. 10.1074/jbc.M110.107821 20353945 PMC2878508

[B16] Koutsoulidou A. Photiades M. Kyriakides T. C. Georgiou K. Prokopi M. Kapnisis K. (2017). Identification of exosomal muscle-specific miRNAs in serum of myotonic dystrophy patients relating to muscle disease progress. *Hum. Mol. Genet.* 26 3285–3302. 10.1093/hmg/ddx212 28637233

[B17] Kramers E.-M. (2015). Physical exercise induces rapid release of small extracellular vesicles into the circulation. *J. Extracell. Vesicles* 1 1–11. 10.3402/jev.v4.28239 26142461 PMC4491306

[B18] Lee E. J. Baek M. Gusev Y. Brackett D. J. Nuovo G. J. Schmittgen T. D. (2008). Systematic evaluation of microRNA processing patterns in tissues, cell lines, and tumors. *RNA* 14 35–42. 10.1261/rna.804508 18025253 PMC2151027

[B19] Lepper C. Partridge T. A. Fan C.-M. (2011). An absolute requirement for Pax7-positive satellite cells in acute injury-induced skeletal muscle regeneration. *Development* 138 3639–3646. 10.1242/dev.067595 21828092 PMC3152922

[B20] Lim L. P. Lau N. C. Garrett-Engele P. Grimson A. Schelter J. M. Castle J. (2005). Microarray analysis shows that some microRNAs downregulate large numbers of target mRNAs. *Nature* 433 769–773. 10.1038/nature03315 15685193

[B21] Lobb R. J. Becker M. Wen S. W. Wong C. S. Wiegmans A. P. Leimgruber A. (2015). Optimized exosome isolation protocol for cell culture supernatant and human plasma. *J. Extracell. Vesicles* 1 1–11. 10.3402/jev.v4.27031 26194179 PMC4507751

[B22] Lugli G. Cohen A. M. Bennett D. A. Shah R. C. Fields C. J. Hernandez A. G. (2015). Plasma exosomal miRNAs in persons with and without Alzheimer disease: altered expression and prospects for biomarkers. *PLoS One* 10:e0139233. 10.1371/journal.pone.0139233 26426747 PMC4591334

[B23] Macaluso F. Isaacs A. W. Di Felice V. Myburgh K. H. (2014). Acute change of titin at mid-sarcomere remains despite 8 wk of plyometric training. *J. Appl. Physiol.* 116 1512–1519. 10.1152/japplphysiol.00420.2013 24458745

[B24] Macaluso F. Myburgh K. H. (2012). Current evidence that exercise can increase the number of adult stem cells. *J. Muscle Res. Cell Motil.* 33 187–198. 10.1007/s10974-012-9302-0 22673936

[B25] Madison R. D. McGee C. Rawson R. Robinson G. A. (2014). Extracellular vesicles from a muscle cell line (C2C12) enhance cell survival and neurite outgrowth of a motor neuron cell line (NSC-34). *J. Extracell. vesicles* 3 1–9. 10.3402/jev.v3.22865 24563732 PMC3930942

[B26] Mccarthy J. J. (2008). MicroRNA-206: the skeletal muscle-specific myomiR. *Biochim. Biophys. Acta* 1779 682–691. 10.1016/j.bbagrm.2008.03.001 18381085 PMC2656394

[B27] McCarthy J. J. (2011). The MyomiR network in skeletal muscle plasticity. *Exerc. Sport Sci. Rev.* 39 150–154. 10.1097/JES.0b013e31821c01e1 21467943 PMC4871711

[B28] Melo S. A. Luecke L. B. Kahlert C. Fernandez A. F. Gammon S. T. Kaye J. (2015). Glypican-1 identifies cancer exosomes and detects early pancreatic cancer. *Nature* 523 177–182. 10.1038/nature14581 26106858 PMC4825698

[B29] Ratajczak M. Z. Ratajczak J. (2016). Horizontal transfer of RNA and proteins between cells by extracellular microvesicles: 14 years later. *Clin. Transl. Med.* 5:7. 10.1186/s40169-016-0087-4 26943717 PMC4779088

[B30] Record M. Carayon K. Poirot M. Silvente-Poirot S. (2014). Exosomes as new vesicular lipid transporters involved in cell-cell communication and various pathophysiologies. *Biochim. Biophys. Acta* 1841 108–120. 10.1016/j.bbalip.2013.10.004 24140720

[B31] Rosenberg M. I. Georges S. A. Asawachaicharn A. Analau E. Tapscott S. J. (2006). Myod inhibits fstl1 and utrn expression by inducing transcription of miR-206. *J. Cell Biol.* 175 77–85. 10.1083/jcb.200603039 17030984 PMC2064500

[B32] Shelke G. V. Lässer C. Gho Y. S. Lötvall J. (2014). Importance of exosome depletion protocols to eliminate functional and RNA-containing extracellular vesicles from fetal bovine serum. *J. Extracell. vesicles* 3 1–8. 10.3402/jev.v3.24783 25317276 PMC4185091

[B33] Skotland T. Ekroos K. Kauhanen D. Simolin H. Seierstad T. Berge V. (2017). Molecular lipid species in urinary exosomes as potential prostate cancer biomarkers. *Eur. J. Cancer* 70 122–132. 10.1016/j.ejca.2016.10.011 27914242

[B34] Small E. M. O’Rourke J. R. Moresi V. Sutherland L. B. McAnally J. Gerard R. D. (2010). Regulation of PI3-kinase/Akt signaling by muscle-enriched microRNA-486. *Proc. Natl. Acad. Sci. U.S.A.* 107 4218–4223. 10.1073/pnas.1000300107 20142475 PMC2840099

[B35] Takov K. Yellon D. M. Davidson S. M. (2017). Confounding factors in vesicle uptake studies using fluorescent lipophilic membrane dyes. *J. Extracell. Vesicles* 6:1388731. 10.1080/20013078.2017.1388731 29184625 PMC5699187

[B36] Uhlemann M. Möbius-Winkler S. Fikenzer S. Adam J. Redlich M. Möhlenkamp S. (2014). Circulating microRNA-126 increases after different forms of endurance exercise in healthy adults. *Eur. J. Prev. Cardiol.* 21 484–491. 10.1177/2047487312467902 23150891

[B37] Van De Vyver M. Myburgh K. H. (2012). Cytokine and satellite cell responses to muscle damage: interpretation and possible confounding factors in human studies. *J. Muscle Res. Cell Motil.* 33 177–185. 10.1007/s10974-012-9303-z 22673937 PMC3413811

[B38] van de Vyver M. Myburgh K. H. (2014). Variable inflammation and intramuscular STAT3 phosphorylation and myeloperoxidase levels after downhill running. *Scand. J. Med. Sci. Sport* 24:e360-71. 10.1111/sms.12164 24383415

[B39] Van Rooij E. Quiat D. Johnson B. A. Sutherland L. B. Qi X. Richardson J. A. (2009). A family of microRNAs encoded by myosin genes governs myosin expression and muscle performance. *Dev. Cell* 17 662–673. 19922871 10.1016/j.devcel.2009.10.013 PMC2796371

[B40] Xie J. X. Fan X. Drummond C. A. Majumder R. Xie Y. Chen T. (2017). MicroRNA profiling in kidney disease: plasma versus plasma- derived exosomes. *Gene* 627 1–8. 10.1016/j.gene.2017.06.003 28587849 PMC5534180

[B41] Yáñez-Mó M. Siljander P. R. Andreu Z. Zavec A. B. Borràs F. E. Buzas E. I. (2015). Biological properties of extracellular vesicles and their physiological functions. *J. Extracell. vesicles* 4:27066. 10.3402/jev.v4.27066 25979354 PMC4433489

